# Steroid therapy and outcome of parapneumonic pleural effusions (STOPPE): Study protocol for a multicenter, double-blinded, placebo-controlled randomized clinical trial

**DOI:** 10.1097/MD.0000000000017397

**Published:** 2019-10-25

**Authors:** Deirdre B. Fitzgerald, Grant W. Waterer, Catherine A. Read, Edward T. Fysh, Ranjan Shrestha, Christopher Stanley, Sanjeevan Muruganandan, Norris S. H. Lan, Natalia D. Popowicz, Carolyn J. Peddle-McIntyre, Najib M. Rahman, Seng Khee Gan, Kevin Murray, Yun Chor Gary Lee

**Affiliations:** a Respiratory Medicine, Sir Charles Gairdner Hospital; b Medical School, Faculty of Health & Medical Sciences; c Pleural Medicine Unit, Institute for Respiratory Health; d Respiratory Medicine, Royal Perth Hospital; e Respiratory Medicine, St John of God Midland; f Respiratory Medicine, Fiona Stanley Hospital; g Respiratory Medicine, Northern Health, Victoria; h School of Allied Health, University of Western Australia; i School of Medical and Health Sciences, Edith Cowan University; j Oxford Respiratory Trials Unit, University of Oxford, UK; k Endocrinology and Diabetes, Royal Perth Hospital, Perth; l School of Population and Global Health, University of Western Australia, Western Australia, Australia.

**Keywords:** clinical stability, corticosteroids, parapneumonic effusion, pleural disease, pneumonia

## Abstract

**Background:**

Community-acquired pneumonia (CAP) is a major global disease. Parapneumonic effusions often complicate CAP and range from uninfected (simple) to infected (complicated) parapneumonic effusions and empyema (pus). CAP patients who have a pleural effusion at presentation are more likely to require hospitalization, have a longer length of stay and higher mortality than those without an effusion. Conventional management of pleural infection, with antibiotics and chest tube drainage, fails in about 30% of cases. Several randomized controlled trials (RCT) have evaluated the use of corticosteroids in CAP and demonstrated some potential benefits. Importantly, steroid use in pneumonia has an acceptable safety profile with no adverse impact on mortality. A RCT focused on pediatric patients with pneumonia and a parapneumonic effusion demonstrated shorter time to recovery. The effects of corticosteroid use on clinical outcomes in adults with parapneumonic effusions have not been tested. We hypothesize that parapneumonic effusions develop from an exaggerated pleural inflammatory response. Treatment with systemic steroids may dampen the inflammation and lead to improved clinical outcomes. The steroid therapy and outcome of parapneumonic pleural effusions (STOPPE) trial will assess the efficacy and safety of systemic corticosteroid as an adjunct therapy in adult patients with CAP and pleural effusions.

**Methods:**

STOPPE is a pilot multicenter, double-blinded, placebo-controlled RCT that will randomize 80 patients with parapneumonic effusions (2:1) to intravenous dexamethasone or placebo, administered twice daily for 48 hours. This exploratory study will capture a wide range of clinically relevant endpoints which have been used in clinical trials of pneumonia and/or pleural infection; including, but not limited to: time to clinical stability, inflammatory markers, quality of life, length of hospital stay, proportion of patients requiring escalation of care (thoracostomy or thoracoscopy), and mortality. Safety will be assessed by monitoring for the incidence of adverse events during the study.

**Discussion:**

STOPPE is the first trial to assess the efficacy and safety profile of systemic corticosteroids in adults with CAP and pleural effusions. This will inform future studies on feasibility and appropriate trial endpoints.

**Trial registration:**

ACTRN12618000947202

**Protocol version:**

version 3.00/26.07.18

## 1 Introduction

Community-acquired pneumonia (CAP) is a major global disease.^[1]^ Pneumonia (including influenza) is currently the 8th leading cause of death in the United States^[2]^ and lower respiratory tract infections were the 3rd leading cause of death worldwide in 2015.^[3]^ CAP incurs significant, immediate, healthcare costs as well as economic costs of time lost from work/school and adverse long-term health problems in survivors.^[4,5]^

Parapneumonic effusions complicate 18% to 57% of cases of pneumonia.^[6–8]^ CAP with an effusion is associated with worse outcomes. In one large study of 4700 patients with CAP, those with an effusion at the time of presentation to the emergency department were more likely to require hospitalization and had a length of stay twice that of those without an effusion.^[9]^ Parapneumonic effusions, inadequately treated, may progress to complicated parapneumonic effusions or empyema, both of which have risen in incidence in recent years.^[10,11]^ Conventional management of pleural infection, with antibiotics and chest tube drainage, fails in about 30% of cases. Many of these patients are frail and unsuitable for surgical management and mortality remains high at 8% in hospital and 20% at 3 months.^[11]^ This mortality rate has increased over the past 2 decades, despite advances in antimicrobial therapy.^[12]^

The pathobiology of pleural effusion formation secondary to pneumonia is not fully understood. One hypothesis is that an over-exaggerated inflammatory response triggered by the infection plays a critical role and may be a target for treatment. Steroids have been shown to be successful adjunct therapies in pneumococcal meningitis and Pneumocystis jirovecii pneumonia, likely due to the significant inflammatory component of these conditions.^[13,14]^ The role of systemic corticosteroids as adjunct treatment in pneumonia is a controversial topic with inconsistent results on mortality from large randomized contolled trials (RCTs).^[15]^ The majority of studies have, however, demonstrated benefits of steroids in shortening time to resolution of fever and clinical stability.^[16–22]^ Importantly, all the studies have demonstrated that use of steroids, even in severe pneumonia, was safe with no adverse impact on mortality.^[23,24]^

The effects of corticosteroid use on clinical outcomes in adults with parapneumonic effusions have not been tested, however, evidence exists in support of the hypothesis that parapneumonic effusions develop from an exaggerated immune response. Patients on long-term inhaled corticosteroids were much less likely to develop a parapneumonic effusion compared with those who were not, and in those who developed a parapneumonic effusion, it was significantly smaller in volume with lower inflammatory indices.^[25]^ Similarly, addition of prednisolone offered faster symptomatic improvement over placebo without major side effects in tuberculosis pleural effusions.^[26]^ Only one study has directly assessed the impact of steroids in parapneumonic effusion. This RCT of pediatric patients found that children treated with dexamethasone infusion had a shorter time to recovery without detrimental impact, except for transient hyperglycemia.^[27]^ These findings strongly support evaluation of the role of adjunct corticosteroid therapy in adults with pneumonia and associated pleural effusions.

The steroid therapy and outcome of parapneumonic pleural effusions (STOPPE) trial is a pilot multicenter, double-blinded, placebo-controlled RCT designed to assess the efficacy and safety of systemic corticosteroid as an adjunct therapy in patients with parapneumonic pleural effusions. The primary hypothesis is that systemic corticosteroids will improve clinical outcome (by reducing pleural and systemic inflammation) in patients with pneumonia-related pleural effusions, and have an acceptable safety profile.

## 2 Methods

### 2.1 Study design and participants

STOPPE is a multicenter, double-blinded, placebo-controlled RCT which will recruit participants with parapneumonic effusion. Participants will be randomized 2:1 to receive intravenous dexamethasone (4 mg twice daily for 48 hours) or placebo. This study will follow participants through their hospital admission and at outpatient visits after 14 days, 30 days, 6 months, and 12 months (Table 1).

**Table 1:**
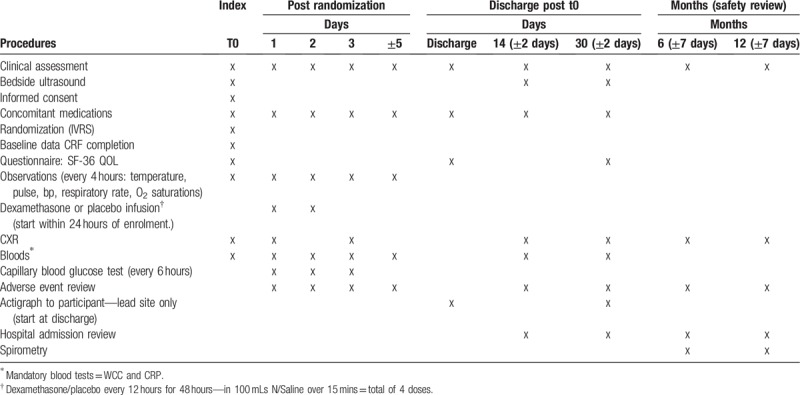
Schedule of enrolment, intervention, data collection, and assessment.

Patients with community-acquired pneumonia will be identified on hospital admission to the General Medicine and Respiratory teams. Those who have a pleural effusion either on admission or develop one within the following 72 hours will be identified. Any patient that meets the inclusion criteria, and none of the exclusion criteria, will be approached by a member of the research team and offered inclusion in the trial. Informed consent will be obtained prior to enrolment. Screening logs will be kept.

Inclusion criteria: New pleural effusion on chest radiography (with bedside ultrasound confirmation) with clinical evidence of a community-acquired pneumonia as per clinician assessment and no alternative causes identified.

Exclusion criteria (similar to those used in recent large multicenter trials of corticosteroid use in pneumonia):

1. Age <18 years;
2. Hemodynamic/respiratory instability requiring intensive care;
3. Acute burn injury;
4. Gastrointestinal bleeding in the last 3 months;
5. Known adrenal insufficiency;
6. Other indication for steroids (e.g., asthma/COPD exacerbation);
7. Long-term steroid use (≥10 mg/d prednisolone equivalent);
8. Acute delirium;
9. Previous steroid-induced psychosis;
10. Severe immunodeficiency (e.g., HIV infection and CD4 count <350 cells/μL), immunosuppressive therapy after organ transplant, leukocytopenia (<1 × 10^9^/L);
11. Cystic fibrosis;
12. Active tuberculosis;
13. Pregnancy/lactation;
14. Un-correctable bleeding diathesis;
15. Poorly-controlled diabetes mellitus (DM);
16. Blood glucose level over 20 mmol/L at the time of screening; and
17. Inability to consent.

### 2.2 Interventions

This trial will randomize participants to receive either dexamethasone 4 mg or placebo administered as an intravenous infusion every 12 hours for 48 hours, that is, to a total of 4 doses. Dexamethasone will be diluted in 100 mL of normal saline and administered via a standard intravenous administration line as a 15-minute infusion. The placebo arm will receive 100 mL of normal saline similarly administered via standard intravenous administration line. Once randomized, the hospital pharmacy will prepare the assigned drug (or placebo). Participants, clinicians, nursing staff, and the research team will be blinded to the treatment arm.

Participants may be withdrawn from the trial if they develop a severe adverse event (SAE). After the identification of a SAE during administration of study drug, dexamethasone/placebo administration may be interrupted for up to 24 hours at the discretion of the local principal investigator and restarted if clinically appropriate to complete the delayed trial treatment course. Participants may also withdraw from the trial at any time if they so wish.

Participants will be able to take their usual medications during the study. Any changes required will be discussed with the participant prior to recruitment. A note of all medications will be taken before the start of the study and will be entered on to the participant's medication log.

### 2.3 Outcomes

As a pilot and first study of corticosteroid in adults with pneumonia-related pleural effusions, we aim to capture a wide range of clinically relevant endpoints which have been used in clinical trials of pneumonia and/or pleural infection, and are considered by clinicians as important. These include:

1. The time to clinical stability defined as the time in hours for all of the following parameters to have been maintained for at least 12 hours: temperature ≤37.4°, heart rate ≤100 bpm, respiratory rate ≤20/min, oxygen saturations ≥95% on room air (unless on home oxygen), systolic blood pressure ≥90 mmHg and tolerating oral intake or discharge home (determined by the treating physician)^[28]^;
2. The time in hours for each of the above parameters to have been maintained for least 12 hours;
3. Change in leukocyte count and C-reactive protein (CRP) over time (Day 0, 1, 3, 14 ± 2 days, 30 ± 2 days);
4. Proportion of participants with a normal leukocyte count and CRP at Day 14 ± 2 and Day 30 ± 2;
5. Improvement in pleural effusion including size of pleural opacity as percentage of hemithorax (as used in our prior studies)^[29]^ over the first 14 ± 2 days and at Day 30 ± 2 compared with baseline; and proportion of participants whose effusion has resolved at day 14 ± 2 days and day 30 ± 2;
6. Proportion of participants requiring an invasive pleural procedure including therapeutic aspirate (volume >200 mL) or chest drain insertion at <48 hours and >48 hours post-commencement of treatment. If intercostal catheter is inserted, the following will be recorded:
  a. Duration of chest tube drainage;
  b. Volume of fluid drained daily;
  c. Proportion treated with intrapleural fibrinolytic therapy; and
  d. Proportion requiring additional drainage procedures including additional chest drain insertion and therapeutic thoracentesis.
7. Proportion of patients referred for thoracic surgery;
8. Duration of antibiotic therapy (intravenous and oral);
9. Length of hospital stay from commencement of treatment to discharge or death;
10. Quality of life measurements including Short form-36 (SF-36) questionnaire^[30]^ and actigraphy (see Section 2.7 for details);
11. Adverse events including but not limited to hyperglycemia, insulin use, need for intensive care, nosocomial infection, gastrointestinal bleeding and re-admission to hospital of any cause; and
12. Overall survival.

### 2.4 Sample size

In the absence of existing data, this pilot study aims to recruit 80 participants over 18 months. This will allow inclusion of the full range of parapneumonic effusions at various stages from simple parapneumonic stage (most common) to empyema (about 5–10% of all cases). The 2:1 randomization strategy will include 50+ participants into the dexamethasone arm to increase the power of detecting treatment related adverse events. The results of this pilot study will inform the power calculation of future studies and determine the most appropriate endpoints of future trials. Past evidence and data suggest that we will be able to estimate a range of outcome parameters with a high enough degree of accuracy with a sample size of 80 and allow some initial comparisons between treatment and placebo. The number of patients included over this timeframe will inform the feasibility and design of further studies.

### 2.5 Randomization and blinding

Participants will be randomly assigned (2:1) to receive intravenous dexamethasone or placebo. The National Health and Medical Research Council Clinical Trials Centre, Sydney, Australia provides the randomization setup via their automated telephone-based interactive voice response services. Randomization is minimized for:

(i) A score of <2 versus ≥2 using the predictive score of Chalmers et al^[6]^ for the development of complicated pleural effusion/empyema secondary to pneumonia (one point each for: serum albumin <30 g/L, CRP >100 mg/L, platelet count >400 × 10^9^/L, serum sodium <130 mmol/L, intravenous drug use, and chronic alcohol excess. One point will be deducted for the presence of COPD);
(ii) Known history of diabetes mellitus (vs not); and
(iii) Size of the effusion on chest radiograph (a grading of ≤2 vs >2 and/or having an intercostal catheter in place).^[31]^

The research team, treating physician, nursing staff, and participant will be blinded to the treatment arm. Only pharmacy staff will receive unblinded randomization information in order to prepare the study drug or placebo. In the event that unblinding is required, a designated member of the lead research team will be contacted and will follow the unblinding SOP.

### 2.6 Data collection

Recruited participants will have their baseline data collected including chest radiograph appearances, blood tests (total leukocyte count, CRP) and clinical observations, as shown in Table 1. These parameters will be repeated through the participant's hospitalization and on discharge. Participants will be monitored for adverse events and those requiring interventional management will be noted. All participants will have a capillary blood glucose test four times a day. Participants will be asked to complete the SF-36 quality of life questionnaire at their date of recruitment, at time of hospital discharge and at the Day 30 follow-up visit. Additional blood tests and imaging will be performed at the discretion of the treating physicians. At the 6 and 12 months follow-up visits data on chest x-ray, spirometry, and adverse events will be collected.

Physical activity patterns will be evaluated by a well-validated triaxial accelerometer (ActiGraph GT3X+, Pensacola, FL). The accelerometer will be worn for a 7-day period post-discharge and also from day 30. Participants will fill an activity log while wearing the accelerometer. This objective measurement of physical function has been studied predominantly in people with malignant disease and is expected to be a useful adjunct in assessment of recovery from pneumonia in the future.^[32]^ Accelerometry will be performed on participants at the lead site only.

Where participants do not attend planned study visits the research staff will contact them at home to determine the reason and book an additional visit if required. If the participant misses a visit due to an admission to the site hospital, the visit will be carried out in the hospital providing the participant is well enough. Where this is not possible and the visit is missed, a File Note will be created and kept in the participant's study file. Study visits are carried out according to the individual participant's visit schedule.

### 2.7 Data management

Data collected will be stored at site on password-protected computers accessible only by the site research staff and will be held in the department where the principal investigator (PI) is based.

Data entered will be checked by a second staff to ensure accuracy. All physical documentation will be stored in a secure environment in line with the Australian Code for the Responsible Conduct of Research for clinical trials and local policy guidelines for research data archiving.

### 2.8 Statistical methods

Data will be analyzed on an intention-to-treat basis. Intergroup comparison will be conducted using Student *t* test or Mann–Whitney *U* rank sum test (for parametric and non-parametric distributions, respectively). Changes in parameters before and after treatment within the same subject will be analyzed using a paired *t* test or Wilcoxon signed rank test. Correlations will be analyzed using Pearson or Spearman tests. Multivariate analyses may be performed if indicated. *P* < .05 will be considered statistically significant in the data analyses.

### 2.9 Data monitoring

The Trial Steering Committee will be responsible for the supervision of the trial in all its aspects. It will be responsible for ensuring the completion of the trial to clinical and ethical standards. The Data Safety Monitoring Committee will ensure the safety of study participants through study procedures, reviewing adverse events and serious adverse events, and consider new data (recently published studies) that may determine the validity of study continuation. All deaths, anticipated or unanticipated, will be discussed with the Data Safety Monitoring Committee (DSMC). The committee determines whether significant benefits or risks have been uncovered which may have an impact on the feasibility and/or ethical conduct of the study. The DSMC will also help to ensure the scientific integrity of the study by reviewing the quality of the data it uses to make its decisions.

All serious and non-serious adverse events (AEs) relating to the study will be fully documented on the appropriate case report forms (CRFs). For each AE, the investigator will provide the onset and end dates, intensity, treatment required, outcome, seriousness and action taken. AE logs will be followed up until resolution. Where AEs are not resolved at study completion this will be noted on the AE log.

### 2.10 Ethics and dissemination

The trial has been approved by the Sir Charles Gairdner and Osborne Park Health Care Group Human Research Ethics Committee. Recruitment centers include Sir Charles Gairdner, Fiona Stanley, St John of God Midland, and Royal Perth Hospitals in Western Australia and Northern Hospital in Victoria—approved through the National Mutual Acceptance scheme. Further sites are expected to join.

The investigators will receive approval from the ethics committee for any amendment to the protocol and ensure it is signed by any patient subsequently entering into the trial and those currently in the study, if affected by the amendment. Results of the clinical trial will be presented at scientific meetings and published in medical journals when the trial is completed.

## 3 Discussion

Parapneumonic effusion causes significant morbidity and mortality worldwide and its incidence continues to rise despite advances in anti-microbial therapy.^[10,11]^ Conventional management with antibiotics and chest tube drainage fails in about 30% of cases. Many of these patients are frail and unsuitable for surgical management and mortality remains high. This highlights the need for further therapeutic options in the treatment of parapneumonic effusions. Halting the progression/development of parapneumonic effusions could represent a novel approach.

Dexamethasone is a corticosteroid with predominantly anti-inflammatory effects when compared with alternative corticosteroids. It has been used in the management of many inflammatory conditions. Most of the side effects of dexamethasone are due to long term use and have been well documented. Reassuringly the recent RCTs using short term corticosteroid in CAP patients found no increased incidence of superinfection or gastrointestinal bleeding in the treatment arms. However, the efficacy and safety profiles of dexamethasone in the treatment of parapneumonic effusions in adults have not been studied.

Therefore, the STOPPE trial was designed as the first step towards assessing the efficacy and safety of systemic corticosteroids as an adjunct therapy in adult patients with parapneumonic pleural effusions. This clinical trial will capture a wide range of clinically relevant endpoints on potential roles of corticosteroids in patients with parapneumonic effusions. The results will inform the design of future studies.

## Author contributions

**Conceptualization:** Deirdre B. Fitzgerald, Grant W. Waterer, Catherine A. Read, Natalia D. Popowicz, Carolyn McIntyre, Najib A. Rahman, Kevin Murray, Yun Chor Gary Lee.

**Data curation:** Deirdre B. Fitzgerald, Catherine A. Read, Edward T. Fysh, Ranjan Shrestha, Christopher Stanley, Sanjeevan Muruganandan, Norris S.H. Lan, Carolyn McIntyre, Yun Chor Gary Lee.

**Formal analysis:** Deirdre B. Fitzgerald, Kevin Murray, Yun Chor Gary Lee.

**Funding acquisition:** Deirdre B. Fitzgerald, Grant W. Waterer, Natalia D. Popowicz, Yun Chor Gary Lee.

**Investigation:** Deirdre B. Fitzgerald, Catherine A. Read, Edward T. Fysh, Ranjan Shrestha, Christopher Stanley, Sanjeevan Muruganandan, Norris S.H. Lan, Yun Chor Gary Lee.

**Methodology:** Deirdre B. Fitzgerald, Grant W. Waterer, Natalia D. Popowicz, Najib A. Rahman, Seng Khee Gan, Yun Chor Gary Lee.

**Project administration:** Deirdre B. Fitzgerald, Catherine A. Read, Yun Chor Gary Lee.

**Resources:** Yun Chor Gary Lee.

**Supervision:** Grant W. Waterer, Najib A. Rahman, Yun Chor Gary Lee.

**Visualization:** Deirdre B. Fitzgerald, Yun Chor Gary Lee.

**Writing – original draft:** Deirdre B. Fitzgerald, Norris S.H. Lan, Yun Chor Gary Lee.

**Writing – review & editing:** Deirdre B. Fitzgerald, Grant W. Waterer, Catherine A. Read, Edward T. Fysh, Ranjan Shrestha, Christopher Stanley, Sanjeevan Muruganandan, Norris S.H. Lan, Natalia D. Popowicz, Carolyn McIntyre, Najib A. Rahman, Seng Khee Gan, Kevin Murray, Yun Chor Gary Lee.

Norris S. H. Lan orcid: 0000-0001-8773-4135.
